# Differential Expression of MicroRNA MiR-145 and MiR-155 Downstream Targets in Oral Cancers Exhibiting Limited Chemotherapy Resistance

**DOI:** 10.3390/ijms25042167

**Published:** 2024-02-10

**Authors:** Conner Belnap, Tyler Divis, Karl Kingsley, Katherine M. Howard

**Affiliations:** 1 Department of Advanced Education in Orthodontic Dentistry, School of Dental Medicine, University of Nevada-Las Vegas, 1700 W. Charleston Boulevard, Las Vegas, NV 89106, USA; belnac1@unlv.nevada.edu; 2 Department of Clinical Sciences, School of Dental Medicine, University of Nevada-Las Vegas, 1700 W. Charleston Boulevard, Las Vegas, NV 89106, USA; divis@unlv.nevada.edu; 3 Department of Biomedical Sciences, School of Dental Medicine, University of Nevada-Las Vegas, 1001 Shadow Lane, Las Vegas, NV 89106, USA; katherine.howard@unlv.edu

**Keywords:** oral cancer, chemotherapy resistance, microRNA expression, biomarkers

## Abstract

New evidence has suggested that non-coding microRNAs play a significant role in mediating and modulating chemotherapy resistance, particularly among oral cancers. One recent study found that the upregulation of miR-145 and the downregulation of miR-155 strongly correlated with a limited chemotherapy resistance to Cisplatin, 5-Fluorouracil, and Paclitaxel, although the mechanism(s) responsible for these observations remain unidentified. Using commercially available cell lines of oral squamous cell carcinoma, RNA was isolated, converted into cDNA, and subsequently screened for the expression of downstream targets of miR-145 and miR-155 using qPCR. These results demonstrated the upregulation of miR-21, miR-125, miR-133, miR-365, miR-720, and miR-1246, as well as the downregulation of miR-140, miR-152, miR-218, miR-221, and miR-224. This screening also confirmed the differential expression and regulation of mir-145 and miR-155 among the cell lines with limited chemotherapy resistance (SCC15). In addition, several downstream targets of *these* specific microRNAs were upregulated by all oral cancer cell lines, such as *MBTD1* and *FSCN1*, or downregulated in all cell lines, such as *CLCN3*, *FLI-1*, *MRTFB*, *DAB*, SRGAP1, and *ABHD17C*. However, three miR-145 downstream targets were identified in the least chemotherapy-resistant cells, exhibiting the differential upregulation of *KCNA4* and *SRGAP2*, as well as the downregulation of *FAM135A*, with this expression pattern not detected in any of the other oral cancer cell lines. These data strongly support that the differential regulation of these three downstream targets may be related to the chemosensitivity of this oral cancer cell line. The potential involvement of these targets must be further investigated to determine how and whether mechanisms of these cellular pathways may be involved in the observed lack of chemotherapy resistance. These data may be important to design targets or treatments to reduce chemotherapy resistance and improve patient treatment outcomes.

## 1. Introduction

Oral cancer remains an important epidemiologic concern worldwide, with more than 350,000 cases diagnosed annually, resulting in nearly 200,000 deaths according to recent estimates through 2018 [1]. These high rates of oral cancer morbidity and mortality may be attributable to numerous factors, although many studies now suggest that the late-stage diagnosis of tumors and the advanced age of patients at the time of diagnosis are among the most impactful variables [2,3]. Although many efforts are being made to foster early detection and diagnosis, it has become evident that treatment will be needed for most of these patients and understanding the factors that determine treatment responsiveness among these tumors becomes ever more critical [4,5].

Oral cancer is complex and often involves multiple treatment modalities including surgical resection, chemotherapy, and radiation treatments [6,7]. Depending upon the size, location, and stage of the tumor, oral cancers may be subject to surgical resection structured to remove the tumor mass along with a small margin of normal tissue immediately surrounding the area of concern [8,9]. These procedures may be followed with either radiation or chemotherapy as the main types of follow-up care administered to these oral cancer patients [5,10].

Chemotherapy for oral cancer typically involves one or more of several well-known treatments, such as Cisplatin, 5-Fluorouracil (5-FU), and Paclitaxel (Taxol) [11,12]. Cisplatin functions as a cytotoxic treatment by binding to DNA within the rapidly dividing cells of the tumor and forming a bond between platinum and the nitrogen atom of guanine or “G”, which interferes with transcription, replication, and DNA repair mechanisms [13,14]. Other treatments such as 5-FU function primarily as antimetabolites, inhibiting the function of the enzyme thymidylate synthase, thereby inhibiting an important step in the process of DNA synthesis in rapidly dividing cells, such as tumor cells [15,16]. In addition, chemotherapy agents such as Taxol function by binding microtubules, inducing mitotic arrest at the spindle assembly checkpoint of cell division or the G2/M transition [17,18,19].

Despite the varied mechanisms of action of these chemotherapy agents, many oral cancers also display significant levels of resistance to one or more of these standard treatments [20,21]. The mechanisms proposed to explain this chemoresistance have been identified as specific allelic variations or genetic mutations that allow for metabolic reprogramming and dysregulation to bypass one or more of the chemotherapy pathways or checkpoints, as outlined previously [22,23]. However, new evidence has now suggested that non-coding microRNAs may also play an alternative and significant role in mediating and modulating chemotherapy resistance, particularly among oral cancers [24,25].

MicroRNAs are small, highly conserved, non-coding RNAs involved in the regulation of gene expression through post-transcriptional mediation, such as mRNA inhibition or negative regulation [26,27]. In fact, many studies have identified microRNA expression profiles related to many types of cancers, including lung, breast, and colorectal cancers [28,29,30]. Moreover, recent systematic reviews have identified microRNA expression profiles more closely associated with oral cancers through large-scale salivary biomarker screening studies [31,32,33,34].

More specifically, systematic reviews and meta-analyses have established microRNA expression profiles for oral cancers including miR-21, miR-31, miR-155, and miR-196 [35,36,37,38]. In addition, many studies have revealed that microRNA expression also functions to mediate chemotherapy resistance among oral cancers [24,39]. For example, increased tumor resistance to Cisplatin has been linked with the upregulation of miR-21, but resistance among oral cancer has also been linked with miR-24, miR-218, and miR-629 upregulation, while miR-15b, miR-27b, and miR-155 upregulation may be associated with decreased resistance to Cisplatin within these same tumors [40,41,42,43,44,45].

Recent work from this group has demonstrated the upregulation of miR-21 and miR-365 among oral cancers, as well as the confirmation of the downregulation of miR-27 among chemoresistant oral cancer cell lines [46,47]. Moreover, this most recent study found that the upregulation of miR-145 and the downregulation of miR-155 were also strongly correlated with a lack of chemotherapy resistance, although the mechanism(s) responsible for this observation remained unidentified [47]. The goal of this current study was to provide an evaluation of these microRNAs and their downstream targets to create a more comprehensive understanding of their potential role in the lack of chemotherapeutic resistance among oral cancers, which could provide new potential treatments and therapies [45,47,48]. This study employed a prospective research design to evaluate commercially available oral cancer cell lines for the lack of chemotherapy resistance and then to subsequently screen for the associated upregulation or downregulation of microRNAs, as well as their most likely validated downstream targets using quantitative polymerase chain reaction (qPCR).

## 2. Results

**Figure 1:**
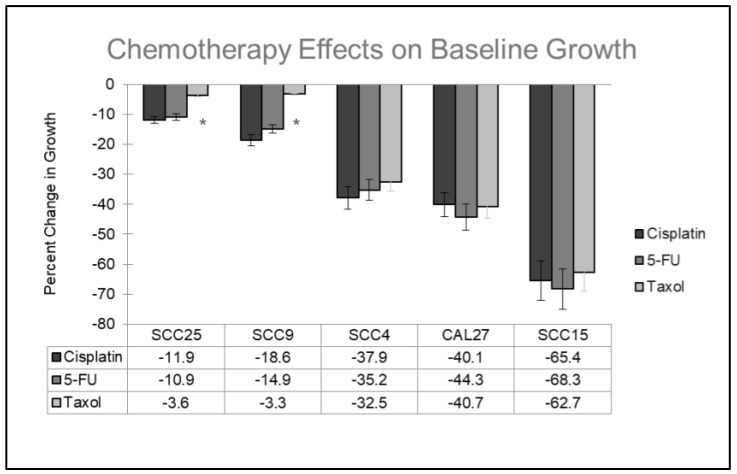
Comparison of baseline (control) growth with experimental treatment among oral cancer cell lines. The administration of all three chemotherapy agents (Cisplatin, 5-FU, Taxol) inhibited growth in all cell lines with the least inhibition observed among SCC25 and SCC9 cells (−3.3% to −18.6%) at concentrations of 1.0, 5.0 and 10.0 ng/mL over three days. Moderate inhibition was observed among SCC4 and CAL27 cells (−32.5% to −44.3%), while the most inhibited (least chemoresistant) cell line was SCC15 (−62.7% to −68.3%). The results were statistically significant (experimental assays compared with baseline growth) except SCC25 (−3.6% *p* = 0.078) and SCC9 (−3.3%, *p* = 0.063) under Taxol administration, denoted by *. Error bars represent standard deviation (SD) derived from three independent experiments, *n* = 8 wells per experimental assay.

The oral cancer cell lines were grown with and without the addition of the chemotherapy agents (Figure 1). More specifically, the addition of Cisplatin, 5-FU, and Taxol inhibited the growth of all oral cancer cell lines at concentrations between 1.0 and 10.0 ng/mL over three days—although these effects exhibited extensive variability. For example, the cell lines SCC25 and SCC9 exhibited the most resistance (and the least inhibition to growth) against all three chemotherapeutic agents, ranging between −3.3% to −18.6% over three days. Other cell lines, such as SCC4 and CAL27, exhibited less resistance and moderate inhibition of cell growth, ranging between −32.5% to −44.3%. However, one cell line in particular, SCC15, exhibited the least resistance to and the most inhibition of growth from all three chemotherapy agents, ranging from −62.7% to −68.3%. All outcomes were statistically significant (experimental assays compared with baseline growth) except SCC25 (−3.6%) and SCC9 (−3.3%) under Taxol administration, *p* = 0.078.

**Figure 2:**
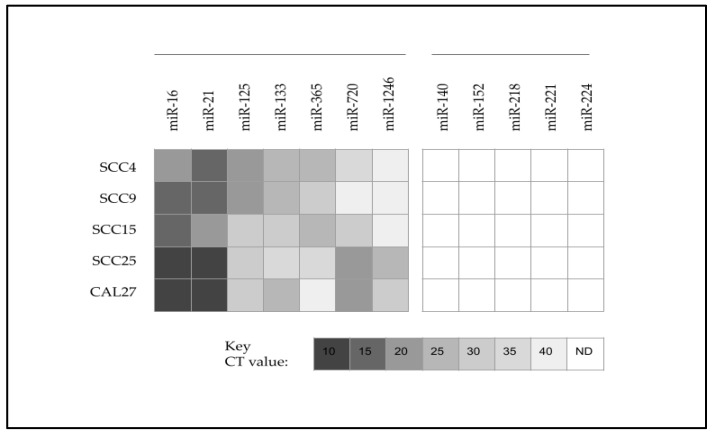
Heatmap analysis of qPCR screening for oral cancer microRNA expression. Oral cancer cell lines upregulated miR-16, miR-21, miR-125, miR-133, miR-365, miR-720, and miR-1246, while downregulation was observed with miR-140, miR-152, miR-218, miR-221, or miR-224. CT = cycle threshold value; N.D. = not detected (below the limit of detection at CT 40).

To confirm and verify the results of the previous studies, RNA was extracted and cDNA was generated (Supplementary File) to facilitate the qPCR screening of microRNAs for all oral cancer cell lines (Figure 2). These results demonstrated that several microRNAs were found to be upregulated in all oral cancers to varying degrees, including miR-16 (positive control), miR-21, miR-125, miR-133, miR-365, miR-720, and miR-1246. In addition, several microRNAs were found to be downregulated (or below the limit of detection at CT 40) among all of the oral cancer cell lines, which included miR-140, miR-152, miR-218, miR-221, and miR-224.

**Figure 3:**
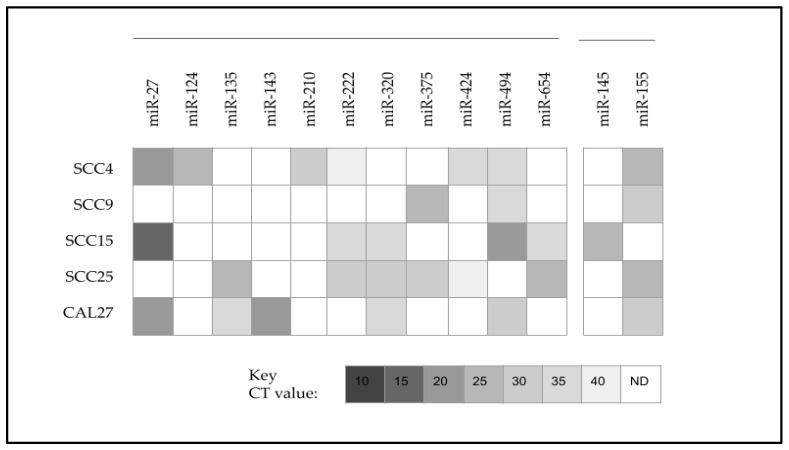
Heatmap analysis of qPCR revealed differential expression of microRNAs among oral cancers. Differentially upregulated microRNAs included miR-27, miR-124, miR-135, miR-143, miR-210, miR-222, miR-320, miR-375, miR-424, miR-494, and miR-654. Differential expression in SCC15 included miR-145 upregulation (only observed among SCC15 cells) and miR-155 downregulation (observed in all other cell lines except SCC15). CT = cycle threshold value; N.D. = not detected (below the limit of detection at CT 40).

Further analysis of the qPCR screening results revealed that several microRNAs were found to be differentially expressed in some, but not all, oral cancer cell lines (Figure 3). For example, miR-124 and miR-210 were upregulated only among SCC4 cells, while miR-143 was upregulated only among CAL27 cells. Most microRNAs were upregulated in at least two or three oral cancer cell lines, including miR-27, miR-135, miR-222, miR-320, miR-375, miR-424 niR-494, and miR-654. However, two microRNAs were differentially expressed between SCC15 and other oral cancer cell lines, which included miR-145, which was only observed among SCC15 cells, and miR-155, which was observed in all other cell lines except SCC15 cells.

**Figure 4:**
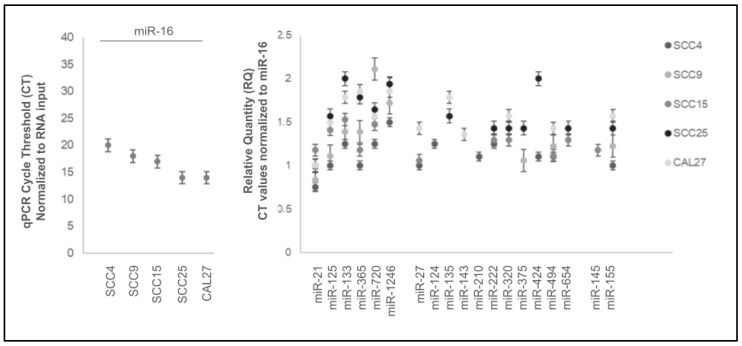
Relative quantification (RQ) of microRNA data. Relative quantity (RQ) normalized to positive control miR-16 CT values was relatively consistent among all cell lines ranging between 0.75 and 2.11, including miR-21 (0.75 to 1.17), miR-125 (1.1 to 1.5), miR-133 (1.25 to 2.0), miR-365 (1.01 to 1.86), miR-720 (1.25 to 2.11), miR-1246 (1.5 to 1.94), miR-27 (1.06 to 1.42), miR-124 (1.25), miR-135 (1.57 to 1.78), miR-143 (1.38), miR-210 (1.11), miR-222 (1.25 to 1.43), miR-320 (1.29 to 1.57), miR-375 (1.06 to 1.42), miR-424 (1.1 to 2.0), miR-494 (1.1 to 1.42), miR-654 (1.29 to 1.43), miR-145 (1.18), and miR-155 (1.22 to 1.57).

Analysis of the microRNA data normalized to the positive control miR-16 revealed that the normalized relative quantity (RQ) for these microRNAs above the limit of detection was relatively consistent (Figure 4). More specifically, the relative quantification of microRNA CT values normalized to miR-16 observed among these cell lines ranged between 0.75 and 2.11, including miR-21 (0.75 to 1.17), miR-125 (1.1 to 1.5), miR-133 (1.25 to 2.0), miR-365 (1.01 to 1.86), miR-720 (1.25 to 2.11), and miR-1246 (1.5 to 1.94). Differentially regulated microRNAs exhibited similar ranges, including miR-27 (1.06 to 1.42), miR-124 (1.25), miR-135 (1.57 to 1.78), miR-143 (1.38), miR-210 (1.11), miR-222 (1.25 to 1.43), miR-320 (1.29 to 1.57), miR-375 (1.06 to 1.42), miR-424 (1.1 to 2.0), miR-494 (1.1 to 1.42), miR-654 (1.29 to 1.43), miR-145 (1.18), and miR-155 (1.22 to 1.57).

**Figure 5:**
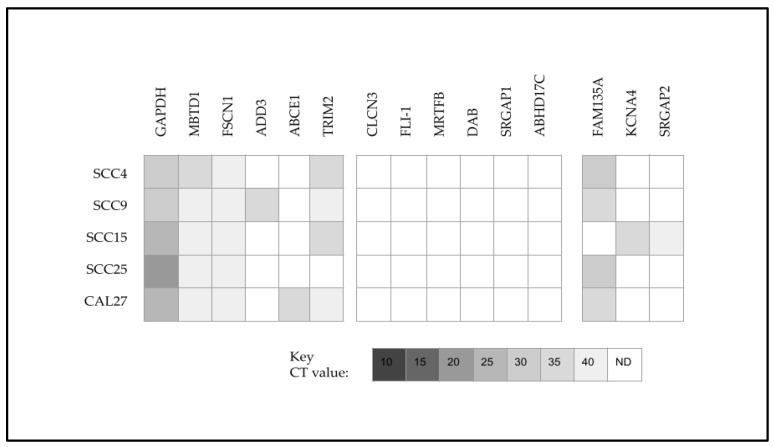
Heatmap of qPCR screening and analysis of miR-145 downstream targets. All cell lines upregulated *MBTD1* and *FSCN1*, while downregulation was observed with *CLCN3*, *FLI*-*1*, *MRTFB*, *DAB*, *SRGAP1*, or *ABHD17C*. Differential expression was observed with *TRIM2*, *ADD3*, and *ABCE1* with SCC15-specific upregulation observed with *KCNA4* and *SRGAP2*, with downregulation observed with *FAM135A*. CT = cycle threshold value; N.D. = not detected (below the limit of detection at CT 40).

To more closely evaluate the potential relationship between miR-145 upregulation and the chemoresistance of SCC15 cells, downstream targets of miR-145 were identified and screened (Figure 5). This analysis revealed that in addition to the positive control *GAPDH*, all oral cancers upregulated the miR-145 downstream targets *MBTD1* and *FSCN1*. In addition, several targets were downregulated (or below the limit of detection at CT40), including *CLCN3*, *FLI-1*, *MRTFB*, *DAB*, *SRGAP1*, and *ABHD17C*. However, differential expression was observed with *TRIM2*, *ADD3*, and *ABCE1* among some of the oral cancer cell lines. Moreover, SCC15-specific upregulation was observed with *KCNA4* and *SRGAP2* and downregulation among SCC15 cells of *FAM135A*, which was observed among all other oral cancer cell lines.

**Figure 6:**
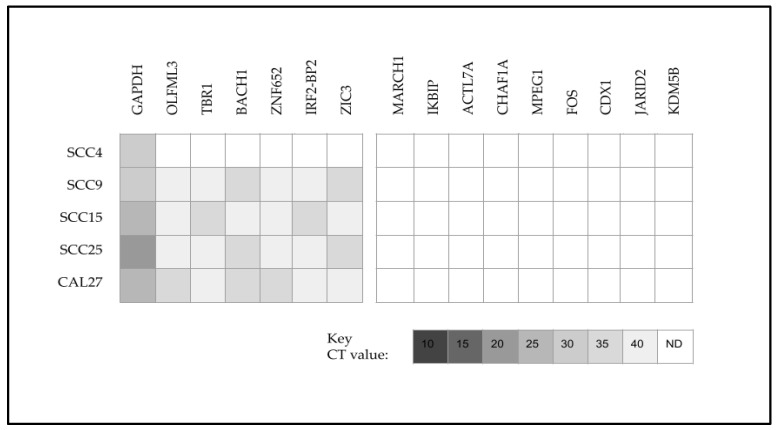
Heatmap of qPCR screening and analysis of miR-155 downstream targets. All cell lines (except SCC4) upregulated *OLFML3*, *TBR1*, *BACH1*, *ZNF652*, *IRF2*-*BP2*, and *ZIC3*, while downregulation was observed with *MARCH1*, *IKBIP*, *ACTL7A*, *CHAF1A*, *MPEG1*, *FOS*, *CDX1*, *JARID2*, and *KDM5B*. No differential or SCC15-specific expression was observed among any of the miR-155 downstream targets analyzed. CT = cycle threshold value; N.D. = not detected (below the limit of detection at CT 40).

To more closely evaluate the potential relationship between the downregulation of miR-155 and the chemoresistance of SCC15 cells, downstream targets of miR-155 were also identified and screened (Figure 6). This analysis revealed that in addition to the positive control *GAPDH*, all oral cancers (except SCC4) upregulated the miR-155 downstream targets *OLFML3*, *TBR1*, *BACH1*, *ZNF652*, *IRF2*-*BP2*, and *ZIC3*. In addition, the oral cancers downregulated *MARCH1*, *IKBIP*, *ACTL7A*, *CHAF1A*, *MPEG1*, *FOS*, *CDX1*, *JARID2*, and *KDM5B*. No differential or SCC15-specific expression was observed among any of the miR-155 downstream targets analyzed.

**Figure 7:**
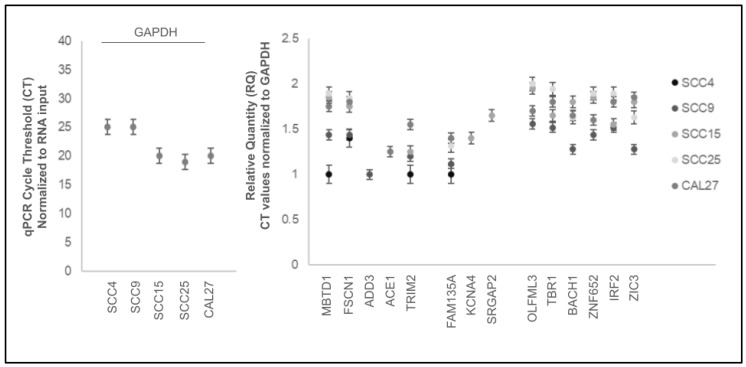
Relative quantification (RQ) of mRNA data. Relative quantity (RQ) normalized to positive control *GAPDH* CT values ranged between 1.11 and 2.01, including miR-145 downstream targets *MBTD1* (1.11 to 1.89), *FSCN1* (1.4 to 1.84), *ADD3* (1.13), *ACE1* (1.25), *TRIM2* (1.15 to 1.55), *FAM135A* (1.12 to 1.4), *KCNA4* (1.4), and *SRGAP2* (1.65) and miR-155 downstream targets *OLFML3* (1.56 to 2.01), *TBR1* (1.52 to 1.95), *BACH1* (1.28 to 1.8), *ZNF652* (1.44 to 1.89), *IRF2* (1.52 to 1.89), and *ZIC3* (1.28 to 1.85).

Analysis of the downstream target data normalized to the positive control *GAPDH* revealed that the normalized relative quantity (RQ) for these mRNAs above the limit of detection was relatively consistent (Figure 7). More specifically, the relative quantification of mRNA CT values normalized to *GAPDH* for the miR-145 downstream targets among these cell lines ranged between 1.11 and 2.01, including *MBTD1* (1.11 to 1.89), *FSCN1* (1.4 to 1.84), *ADD3* (1.13), *ACE1* (1.25), *TRIM2* (1.15 to 1.55), *FAM135A* (1.12 to 1.4), *KCNA4* (1.4) and *SRGAP2* (1.65). Similarly, downstream targets for miR-155 included *OLFML3* (1.56 to 2.01), *TBR1* (1.52 to 1.95), *BACH1* (1.28 to 1.8), *ZNF652* (1.44 to 1.89), *IRF2* (1.52 to 1.89), and *ZIC3* (1.28 to 1.85). 

## 3. Discussion

The primary objective of this current study was to provide an evaluation of the specific microRNA expression profile of the oral cancer cell line SCC15 lacking chemotherapeutic resistance, which may provide new potential insights into potential treatments and therapies. These results confirmed the upregulation of miR-145 among this chemosensitive cell line previously reported by this group [47]. This supports other research that demonstrates that the downregulation of miR-145 expression was associated with oral cancer diagnosis and progression, while miR-145 upregulation correlated with improved prognosis and increased survival [31,39].

In fact, previous research has demonstrated that higher levels of miR-145 are negatively correlated with oral cancer progression and may, in fact, function as an intermediary tumor suppressor [49,50,51]. Some evidence has suggested that miR-145 may function as a primary, direct tumor suppressor in other cancers and may function similarly to inhibit c-myc and CDK6 in oral cancers [52,53]. These mechanisms appear to support many other studies that have demonstrated that the downregulation of miR-145 was associated with oral cancer progression both in vitro and in vivo [54,55].

The importance of miR-145 suppression becomes apparent as more and more overlapping mechanisms to suppress miR-145 activity are discovered, including the activity of circular RNAs, such as circ_ZNF236, circ_005063, and circ_000199 [56,57]. This research has demonstrated that other circular RNAs such as circ_GOLPH3 and circ_0001461 function to inhibit miR-145 as well as to inhibit additional downstream targets, such as KDM2 and NFkB [58,59]. Finally, many other circular RNAs, including circ_0058063 and circ_0033144, may function in concert with additional axis factors to inhibit miR-145 while upregulating other downstream targets, such as SERPINE1 and LASP1 [60,61].

The results of this current study may be the first to demonstrate the upregulation of miR-145 correlated with the downregulation of the predicted downstream target FAM135A, which was observed within the other chemoresistant cell lines and has been recently demonstrated to function in lipid metabolism within other cancers such as breast and pancreatic cancers [62,63]. Moreover, this study also demonstrated the association between miR-145 upregulation and positive expression of the potassium voltage-gated channel protein KCNA4, which was also recently identified in a genome-wide differential expression study of renal cell carcinomas [64]. Finally, this study found that miR-145 upregulation was positively associated with SRGAP2 expression, which is a Rho GTPase-activating protein originally identified as regulating neuronal migration and differentiation but more recently identified as a potential chemoregulatory modulator in hepatocellular carcinomas and colorectal cancers [65,66,67].

Although this study found no downstream targets of miR-155 that were differentially expressed, it is clear that the downregulation of miR-155 among the chemosensitive cell line SCC15 is significant, as this has been identified by other studies as a direct activator of additional downstream targets, such as the anti-apoptosis regulator BCL6 and pro-cell cycle regulator Cyclin D2 [68,69]. In addition, many studies have confirmed that miR-155 upregulation may directly contribute to chemotherapy resistance to 5-FU and Cisplatin among oral cancers through additional pathway modulation, such as TP53INP1 [70,71,72]. Thus, continued research to confirm the downregulation of miR-155 among chemosensitive oral cancers may also help the understanding and delineation of which factors may be critical for designing treatments and therapies that increase effectiveness and efficacy.

Although these findings are significant, there are some limitations associated with this study, which should be addressed for further consideration. First, this study employed well-characterized, commercially available cell lines of oral cancers to create a model system for analyzing and evaluating chemoresistance and chemosensitivity, which may not reflect the diversity or range of expression profiles among current patients with oral cancer. However, this type of *in vitro* validation system has been used in multiple research studies that sought to analyze and evaluate types of cancer-related chemotherapy resistance and sensitivity, as well as the mechanisms that may be operational in these systems [47,71,73]. Although this type of research is essential to uncover chemoresistance and chemosensitivity and the underlying mechanisms that may be responsible, these results must be analyzed and validated in future studies that utilize primary tissue samples from oral cancer patients and a wider range of tumor explants [74,75]. Finally, more studies of this nature will be needed to carefully evaluate how to select and screen for the downstream microRNA targets identified in various databases, which could help to design and develop new types of treatments and therapies based upon these discoveries [76,77].

## 4. Materials and Methods

### 4.1. Cell Lines and Culture

This study utilized commercial oral cancer cell lines, which included oral squamous cell carcinomas (OSCC) of the tongue. All cell lines were purchased from the American Tissue Culture Collection or ATCC (Manassas, VA, USA). These included SCC4, SCC9, SCC15, SCC25, and CAL27. All cells were cultured and maintained using the protocols and recommendations from the manufacturer. In brief, Dulbecco’s Modified Eagle’s Medium (DMEM) supplemented with fetal bovine serum or FBS (10%) and antibiotic Penicillin–Streptomycin (1%), all from Fisher Scientific (Fair Lawn, NJ, USA), were used for CAL27 cells. All other cell lines (SCC25, SCC15, SCC9, SCC4) were maintained using DMEM:F12 with 10% FBS and 1% Penicillin–Streptomycin, all obtained from Fisher Scientific (Fair Lawn, NJ, USA). Catalog information for ordering, the short tandem repeat (STR) analysis for the verification of cell type (>90%), and the original derivation of each cell line provided by the manufacturer are provided as follows (Table 1):

The culture of cells was facilitated using tissue culture-treated flasks and a FisherBrand Isotemp CO_2_ Biosafety Level 2 (BSL-2) incubator from Fisher Scientific (Fair Lawn, NJ, USA) at 37 °C, which was supplemented with additional medical-grade CO_2_ at 5%.

### 4.2. Experimental Chemotherapy Agents

Experimental assays utilized commercially available chemotherapy agents, which included Paclitaxel (NSC 125973, Molecular Weight 853.91), 5-Fluorouracil (NSC 19893, Molecular Weight 130.08), and cis-diamminedichloroplatinum (Cisplatin; NSC 119875, Molecular Weight 300.5), all obtained from Selleck Chemical (Houston, TX, USA). Concentrations for each chemotherapy agent used in the proliferation and growth assays were within the range of 1.0, 5.0, and 10.0 ng/mL to simulate the low, mid, and high physiologic concentrations and dosages that have been validated through previous *in vivo* bioavailability studies [78,79].

### 4.3. Proliferation Assays and Statistical Analyses

Oral cancer cell growth under experimental (chemotherapy) and control (no treatment) conditions was performed using Corning Costar 96-well tissue culture-treated assay plates from Fisher Scientific (Fair Lawn, NJ, USA). Cells were plated at standard concentrations (1 × 10^5^ cells/mL) and were allowed to proliferate for 24, 48, and 72 h with and without chemotherapeutic agents to establish baseline growth and determine chemotherapeutic inhibition for each cell line. All assays were performed in triplicate and each assay was performed using n = 8 wells per cell line and condition. At the conclusion of each endpoint (24 h, 48 h, 72 h), cells were fixed using 10% buffered formalin prior to processing. The processing of each assay plate was performed by removing the buffered formalin and adding Gentian Violet 1% aqueous solution from Ricca Chemicals (Arlington, TX, USA). The stain was aspirated and wells were washed with 10% phosphate-buffered saline (PBS) obtained from Fisher Scientific (Fair Lawn, NJ, USA). All liquid was aspirated and plates were analyzed using an ELx808 Microplate Reader from BioTek Instruments (Winsooki, VT, USA) at 630 nm.

Statistical analyses of differences between baseline (control, no chemotherapy) and experimental assays were completed using two-tailed Student’s *t*-tests and an alpha level of 0.05 for statistical significance. With each assay performed in triplicate using n = 8 wells per cell line and experimental condition, a total of n = 24 data points were used for each analysis.

### 4.4. RNA Extraction

Cellular RNA was extracted from all cell lines for further screening and analysis. This process involved the phenol:chloroform extraction method, utilizing the TRIzol reagent obtained from Invitrogen (Waltham, MA, USA) according to the manufacturer’s recommended protocol. In brief, the supernatant was removed from cells in culture and TRIzol reagent was added to facilitate cell lysis. To each cellular lysate, molecular-grade chloroform from Invitrogen (Waltham, MA, USA) was added and mixed prior to incubation and centrifugation. The upper aqueous layer containing RNA was then transferred and molecular-grade isopropanol was added to precipitate nucleic acids. Each sample was then centrifuged to pellet the nucleic acids. The isopropanol was removed and the nucleic acid-containing pellet was washed with ethanol and centrifuged again. Each pellet was then resuspended using nuclease-free water. Assessments of RNA concentrations and quality were performed using the NanoDrop 2000 Spectrophotometer obtained from Fisher Scientific (Fair Lawn, NJ, USA). Relative quantification and purity were determined using absorbance readings at A260 nm and A280 nm.

RNA was converted for qPCR screening with the ABgene Reverse-iT One-Step RT-PCR kit from Fisher Scientific (Fair Lawn, NJ, USA). Reddy Mix RT-PCR Master mix and RTase blend from this kit were combined with the total extracted RNA (1.0 ug) in addition to universal forward and reverse random primers by Invitrogen (Waltham, MA, USA) according to the manufacturer’s protocol. cDNA synthesis reactions were performed using a Mastercycler gradient thermal cycler from Eppendorf (Hamburg, Germany) using the manufacturer recommendation protocol of reverse transcription at 47 °C for 30 min and a final extension for five minutes at 72 °C. Analysis of cDNA was completed with a NanoDrop 2000 Spectrophotometer (Fisher Scientific; Fair Lawn, NJ, USA), and absorbance readings were taken at A260 and A280 nm.

### 4.5. microRNA cDNA Synthesis

To amplify low-volume microRNA from the oral cancer cell lines, RNA was processed using the TaqMan Advanced miRNA Assay conversion kit from Applied Biosystems (Waltham, MA, USA), as previously described [47,75]. This protocol includes a poly-adenylation reaction, which involved 0.5 µL 10× poly(A) buffer, 0.5 µL ATP (adenosine triphosphate), 0.3 µL poly(A) enzyme, and 1.7 µL RNase-free water added to each of the 96-wells in a qPCR reaction plate with 2.0 µL of RNA extracted from each cell line. The poly-adenylation reaction was performed using the manufacturer’s recommended protocol of 37 °C for 45 min followed by 65 °C for 10 min in a Mastercycler gradient thermal cycler from Eppendorf (Hamburg, Germany).

Following the completion of the poly-adenylation reaction, the adaptor ligation reaction was immediately performed using 3.0 µL 5× DNA ligase buffer, 4.5 µL RNase-free water added to each of 50% PEG (polyethylene glycol) 8000, 0.6 µL 25× ligation adaptor, 1.5 µL RNA ligase, and 0.4 µL RNase-free water added to each of the 96-wells containing the completed poly-adenylation reaction in the qPCR reaction plate. The adaptor ligation reaction was performed using the manufacturer’s recommended protocol of 16 °C for 60 min.

Following the adaptor ligation reaction, the reverse transcription (RT) reaction was immediately performed using 6.0 µL 5× RT buffer, 1.2 µL dNTP mix, 1.5 µL 20× universal RT primer, 3.0 µL 10 × RT enzyme mix, and 3.3 µL RNase-free water added to each of the 96-wells containing the adaptor ligation reaction. The RT reaction was performed using the manufacturer’s recommended protocol of 42 °C for 15 min, followed by 85 °C for an additional five minutes.

The final reaction step in this protocol was the amplification of the cDNA using the TaqMan miR-Amp Reaction Mix, which included 25.0 µL 2× miR-Amp Master Mix, 2.5 µL 20× Primer Mix and nuclease, 17.5 µLRNase-free water, and 5.0 uL of the RT reaction product. The amplification reaction was performed using the manufacturer’s recommended protocol of 95 °C for five minutes, followed by 14 cycles of 95 °C for three seconds, annealing and extension at 60 °C for 30 s, and a stop reaction at 99 °C for ten minutes.

### 4.6. qPCR Screening

Screening of the cDNA for microRNA expression was completed using the SYBR Green qPCR Master Mix from ThermoFisher Scientific (Fair Lawn, NJ, USA) using the manufacturer’s recommended protocols. In brief, each reaction was prepared with 12.5 uL Absolute SYBR Green, 1.75 uL forward and reverse primers, 7.5 uL nuclease-free water, and 1.5 uL sample cDNA for a total reaction volume of 25 uL. Thermocycle reactions were performed using the QuantStudio Real-Time Polymerase Chain Reaction (PCR) system from Applied Biosciences (Waltham, MA, USA) with 95 °C denaturation for 15 s, annealing at each primer pair’s specific temperature, and a 72 °C final extension for 30 s. Positive control primers included miR-16, which was used as the reference standard for the normalization of microRNA targets, and glyceraldehyde 3-phosphate dehydrogenase (GAPDH), which was used as a reference gene for the normalization of the downstream mRNA targets.

The downstream microRNA targets were selected using the MicroRNA Target Prediction Database (www.mirdb.org). The predicted miR-145 with the highest target scores (above 90) that were validated from previous studies in other model systems included *FSCN1*, *ABHD17C*, *FLI-1*, *MRTFB, DAB2, SRGAP1, SRGAP2, CLCN3, MBTD1, FAM135A, ABCE1, KCNA4, ADD3,* and *TRIM2*. The predicted miR-155 targets with the highest target scores (above 90) that were validated from previous studies in other model systems included *ZNF652, ZIC3, BACH1, JARID2, KDM5B, TBR1, IRF2, OLFML3, MPEG1, CDX1, ACTL7A, MARCH1, FOS, IKBIP,* and *CHAF1A*. The validated primer sets (Table 2) included [45,80]:

## 5. Conclusions

The main objective of this study was to determine if microRNAs or their downstream targets were differentially expressed among chemosensitive oral cancer cells. These results provide evidence and support that the differential regulation of key microRNAs, such as miR-145 upregulation and miR-155 downregulation, may be specifically associated with the chemosensitive SCC15 oral cancer cell line. In addition, the identification of differential expression and the potential involvement of validated downstream targets specific to miR-145, including *FAM135A*, *KCNA4*, and *SRGAP2* may therefore allow for further investigation to determine how and whether mechanisms involving these cellular molecules may be responsible for the observed lack of chemotherapy resistance. These data may be important to design future targets, therapies, or treatments to reduce oral cancer chemotherapy resistance and improve future patient treatment outcomes.

## Figures and Tables

**Table 1:** Cell line information and manufacturer analysis.

Cell Line	Media	Designation Cell Type	STR Analysis	Sex	Age
CAL27 (CRL-2095)	DMEM	OSCC	93%	Male	56 years
SCC25 (CRL-1628)	DMEM:F12	OSCC	100%	Male	70 years
SCC15 (CRL-1623)	DMEM:F12	OSCC	95%	Male	55 years
SCC9 (CRL-1629)	DMEM:F12	OSCC	100%	Male	25 years
SCC4 (CRL-1624)	DMEM:F12	OSCC	92%	Male	55 years

**Table 2:** Validated qPCR primer sets.

Control primers	Primer sequence	Temp.
GAPDH forward	5′ATCTTCCAGGAGCGAGATCC-3′	Tm: 66 °C
GAPDH reverse	5′ACCACTGACACGTTGGCAGT-3′	Tm: 70 °C
Beta-actin forward	5′-GTGGGGTCCTGTGGTGTG-3′	Tm: 69 °C
Beta-actin reverse	5′-GAAGGGGACAGGCAGTGA-3′	Tm: 67 °C
miRNA primers	Primer sequence	Temp.
miR-16 forward	5′-TAGCAGCACGTAAATATTGGCG-3	Tm: 65 °C
miR-16 reverse	5′-TGCGTGTCGTGGAGTC-3′	Tm: 65 °C
miR-21 forward	5′-GCCACCACACCAGCTAATTT-3′	Tm: 66 °C
miR-21 reverse	5′-CTGAAGTCGCCATGCAGATA-3′	Tm: 65 °C
miR-27 forward	5′-ATATGAGAAAAGAGCTTCCCTGTG-3′	Tm: 61 °C
miR-27 reverse	5′-CAAGGCCAGAGGAGGTGAG-3′	Tm: 67 °C
miR-124 forward	5′-TTCACAGCGGACCTTGA-3′	Tm: 64 °C
miR-124 reverse	5′-GAACATGTCTGCGTATCTC-3′	Tm: 60 °C
miR-125 forward	5′-GCCCTCCCTGAGACCTCAA-3′	Tm: 69 °C
miR-125 reverse	5′-GTGCAGGGTCCGAGGT-3′	Tm: 68 °C
miR-133 forward	5′-CCGGTTAACTCGAGCTCTGTGAGAG-3′	Tm: 71 °C
miR-133 reverse	5′-CTAGCTAGGAATTCTGTGACCTGTG-’3′	Tm: 66 °C
miR-135 forward	5′-CGATATGGCTTTTTATTCCTA -3′	Tm: 56 °C
miR-135 reverse	5′-GAGCAGGGTCCGAGGT -3′	Tm: 67 °C
miR-140 forward	5′-GGGCAGTGGTTTTACCCTA -3′	Tm: 64 °C
miR-140 reverse	5′-CAGTGCGTGTCGTGGAGT -3′	Tm: 68 °C
miR-143 forward	5′-AGTGCGTGTCGTGGAGTC-3′	Tm: 68 °C
miR-143 reverse	5′-GCCTGAGATGAAGCACTGT-3′	Tm: 65 °C
miR-145 forward	5′-AGAGAACTCCAGCTG-3′	Tm: 56 °C
miR-145 reverse	5′-GGCAACTGTGGGGTG-3′	Tm: 64 °C
miR-152 forward	5′-GGTTCAAGACAGTACGTGACT-3′	Tm: 64 °C
miR-152 reverse	5′-CCAAGTTCTGTATGCACTGA-3′	Tm: 62 °C
miR-155 forward	5′-TTAATGCTAATTGTGATAGGGGT-3′	Tm: 61 °C
miR-155 reverse	5′-CCTATCACAATTAGCATTAATT-3′	Tm: 55 °C
miR-210 forward	5′-CATAGATAGCCACTGCCCACA-3′	Tm: 67 °C
miR-210 reverse	5′-GTGCAGGGTCCGAGGTATTC-3′	Tm: 68 °C
miR-218 forward	5′-TCGGGCTTGTGCTTGATC T-3′	Tm: 65 °C
miR-218 reverse	5′-GTGCAGGGTCCGAGTG-3′	Tm: 66 °C
miR-221 forward	5′-CCCAGCATTTCTGACTGTTG-3′	Tm: 64 °C
miR-221 reverse	5′-TGTGAGACCATTTGGGTGAA-3′	Tm: 64 °C
miR-222 forward	5′-CGCAGCTACATCTGGCTACTG-3′	Tm: 68 °C
miR-222 reverse	5′-GTGCAGGGTCCGAGGT-3′	Tm: 68 °C
miR-224 forward	5′-GCGAGGTCAAGTCACTAGTGGT-3′	Tm: 69 °C
miR-224 reverse	5′-CGAGAAGCTTGCATCACCAGAGAACG-3′	Tm: 72 °C
miR-320 forward	5′-AACGGAGAGTTGGGTCGAAA-3′	Tm: 66 °C
miR-320 reverse	5′-TTGCCTCTCAACCCAGCTTT-3′	Tm: 67 °C
miR-365 forward	5′-ATAGGATCCTGAGGTCCCTTTCGTG-3′	Tm: 70 °C
miR-365 reverse	5′-GCGAAGCTTAAAAACAGCGGAAGAGTTT-3′	Tm: 72 °C
miR-375 forward	5′-GGCTCTAGAGGGGACGAAGC-3′	Tm: 70 °C
miR-375 reverse	5′-GGCAAGCTTTTTCCACACCTCAGCCTTG-3′	Tm: 74 °C
miR-424 forward	5′-AGGACGAAACACCCCCTATTCCTTGC-3′	Tm: 73 °C
miR-424 reverse	5′-TAATGGATCCGAATACCTGCTCCTGA-3′	Tm: 69 °C
miR-494 forward	5′-GAAGATCTACGTCTGGTCTACCCAGTGC-3′	Tm: 72 °C
miR-494 reverse	5′-GGGGTACCACCGAGAGTGGAGCCGGCAA-3′	Tm: 82 °C
miR-654 forward	5′-GGGATGTCTGCTGACCA-3′	Tm: 64 °C
miR-654 reverse	5′-CAGTGCGTGTCGTGGA-3′	Tm: 65 °C
miR-720 forward	5′-GCGTGCTCTCGCTGGGG-3′	Tm: 73 °C
miR-720 reverse	5′-GTGCAGGGTCCGAGGT-3′	Tm: 68 °C
miR-1246 forward	5′-TGAAGTAGGACTGGGCAGAGA-3′	Tm: 67 °C
miR-1246 reverse	5′-TTTGGGTCAGGTGTCCACTC-3′	Tm: 67 °C
miR-145 targets	Primer sequence	Temp.
FSCN1 forward	5′-CCAGGGTATGGACCTGTCTG-3′	Tm: 65 °C
FSCN1 reverse	5′-GTGTGGGTACGGAAGGCAC-3′	Tm: 65 °C
ABHD17C forward	5′-CTACTCGGGATACGGCGTCA-3′	Tm: 65 °C
ABHD17C reverse	5′-AGAGGATAATGTTCTCGGGACTC-3′	Tm: 63 °C
FLI forward	5′-CAGCCCCACAAGATCAACCC-3′	Tm: 65 °C
FLI reverse	5′-CACCGGAGACTCCCTGGAT-3′	Tm: 65 °C
MRTFB forward	5′-ATGGATCACACAGGGGCGATA-3	Tm: 63 °C
MRTFB reverse	5′-CCGCTGGGCTCTTCAAAGG-3′	Tm: 65 °C
DAB2 forward	5′-GTAGAAACAAGTGCAACCAATGG-3′	Tm: 61 °C
DAB2 reverse	5′-GCCTTTGAACCTTGCTAAGAGA-3′	Tm: 61 °C
SRGAP1 forward	5′-ACCCCGAGCCGATTCAAGA-3′	Tm: 62 °C
SRGAP1 reverse	5′-GAACTCGCATCTCCGTTTGCT-3′	Tm: 63 °C
SRGAP2 forward	5′-TGAAGGAGAAAGCGTCAAGCC-3′	Tm: 62 °C
SRGAP2 reverse	5′-AAGGTCAGATAGGTCATGGATGT-3′	Tm: 61 °C
CLCN3 forward	5′-GGAGGCAGCATTAACAGTTCT-3′	Tm: 61 °C
CLCN3 reverse	5′-TCGCACCCAATCAATAGTATGGA-3′	Tm: 61 °C
MBTD1 forward	5′-GGCATGGCTACCTGTGAGATG-3′	Tm: 65 °C
MBTD1 reverse	5′-GGCCAAAATGCTTGCCTTCT-3′	Tm: 61 °C
FAM135A forward	5′-AGTAGCCGAACATTGAAGCTG-3′	Tm: 61 °C
FAM135A reverse	5′-TGGCTGGTGTAGTGCAACC-3′	Tm: 62 °C
ABCE1 forward	5′-GGAATGCAAAAAGAGTTGTCCTG-3′	Tm: 61 °C
ABCE1 reverse	5′-CGAGGGATAGGCAACCTGTG-3′	Tm: 65 °C
KCNA4 forward	5′-GTACCTCCCATGACCCTCAGA-3′	Tm: 65 °C
KCNA4 reverse	5′-CTGCCGGTAGTGGGCTTTC-3′	Tm: 65 °C
ADD3 forward	5′-CCAGCCAAGGCGTGATTAC′	Tm: 62 °C
ADD3 reverse	5′-TGAAGTCTTGTCGTAGATCAGGA-3′	Tm: 61 °C
TRIM2 forward	5′-TGCGCCAGATTGACAAGCA′;	Tm: 60 °C
TRIM2 reverse	5′-GCACCTCTCGCAGAAAGTG-3′	Tm: 62 °C
miR-155 targets	Primer sequence	Temp.
ZNF652 forward	5′-GCTGGTTGAAAACTGTGCTGT-3′	Tm: 61 °C
ZNF652 reverse	5′-GAAGATGGCACTTGACCACGA-3′	Tm: 63 °C
ZIC3 forward	5′-CGGCGCACGATCTATCTTCAG-3′	Tm: 65 °C
ZIC3 reverse	5′-TGCGGAACAGAAACTCGC-3′	Tm: 62 °C
BACH1 forward	5′-TCTGAGTGAGAACTCGGTTTTTG-3′	Tm: 61 °C
BACH1 reverse	5′-CGCTGGTCATTAAGGCTGAGTCC-3′	Tm: 63 °C
JARID2 forward	5′-ACCAGTCTAAGGGATTAGGACC-3′	Tm: 63 °C
JARID2 reverse	5′-TGCTGGGACTATTCGGCTGA-3′	Tm: 62 °C
KDM5B forward	5′-CCATAGCCGAGCAGACTGG-3′	Tm: 65 °C
KDM5B reverse	5′-GGATACGTGGCGTAAAATGAAGT-3′	Tm: 61 °C
TBR1 forward	5′-GCAGCAGCTACCCACATTCA-3′	Tm: 62 °C
TBR1 reverse	5′-AGGTTGTCAGTGGTCGAGATA-3′	Tm: 61 °C
IRF2-BP2 forward	5′-CCCATGACTCCTACATCCTCTT-3′	Tm: 63 °C
IRF2-BP2 reverse	5′-GAGGGCGGACTGTTGCTATTC-3′	Tm: 65 °C
OLFML3 forward	5′-TCCTTTTGTCATGGTCGGGAC-3′	Tm: 63 °C
OLFML3 reverse	5′-TAAAGCAGCTAGTCGGCGTTC-3′	Tm: 63 °C
MPEG1 forward	5′-CGGCAGCATGGGCTAAATCA-3′	Tm: 62 °C
MPEG1 reverse	5′-TGTCCACATTCCGCAGATTGT-3′	Tm: 61 °C
CDX1 forward	5′-GGTGGCAGCGGTAAGACTC-3′	Tm: 65 °C
CDX1 reverse	5′-TGTAACGGCTGTAATGAAACTCC-3′	Tm: 61 °C
ACTL7A forward	5′-TGGGTCCGCCATACGAGTT-3′	Tm: 62 °C
ACTL7A reverse	5′-GTCCACGACCACTGCTTTG-3′	Tm: 62 °C
MARCH1 forward	5′-CACTGGGACACTGCGCTTT-3′	Tm: 62 °C
MARCH1 reverse	5′-TCACAGCAGCGTGTATCTGAG-3′	Tm: 63 °C
FOS forward	5′-CCGGGGATAGCCTCTCTTACT-3	Tm: 65 °C
FOS reverse	5′-CCAGGTCCGTGCAGAAGTC-3′	Tm: 65 °C
IKBIP forward	5′-GCTCATCTAAAGCGTCTACAGG-3′	Tm: 63 °C
IKBIP reverse	5′-AAGCGTCGTCAGACTGTTGTT-3′	Tm: 61 °C
CHAF1A forward	5′-AGCCCGTCTGCCGTTTAAG-3′	Tm: 62 °C
CHAF1A reverse	5′-AGAAGTACCCTGATCGTCTGAC-3′	Tm: 63 °C

## Data Availability

The data presented in this study are available on request from the corresponding author.

## Supplementary Materials

The following supporting information can be downloaded at: https://www.mdpi.com/article/10.3390/ijms25042167/s1.
