# Supplementary material for: Differential Expression of MicroRNA MiR-145 and MiR-155 Downstream Targets in Oral Cancers Exhibiting Limited Chemotherapy Resistance
Source: Int J Mol Sci. 2024 Feb 10;25(4):2167. doi: 10.3390/ijms25042167 (PMC10889714; doi:10.3390/ijms25042167)
Supplement: Supplementary file 1 [file ijms-25-02167-s001.zip › ijms-2843325-supplementary.pdf]

To determine any differences in expression that may modulate the observed differences in chemotherapy resistance, RNA was extracted from all cell lines (Supplementary Table). These data demonstrated the successful isolation of RNA from all cell lines, which averaged 454.6 +/- 44.6 ng/uL and ranged from 422 to 492 ng/uL. Purity of RNA, determined by the ratio of absorbance at A260 to A280, averaged 1.79 among the cancer cell lines with a range between 1.77 and 1.81. Synthesis of cDNA from the isolated RNA was completed, which demonstrated concentrations that averaged 1526 +/- 53.6 with a range between 1499 and 1552 ng/uL. Purity of cDNA averaged 1.84, which ranged between 1.81 and 1.88.

Supplementary Table . RNA and cDNA analysis.

**Cell Line**	**RNA Concentration [ng/uL]**	**cDNA Concentration**	**Purity A260:A280**
SCC4	422 +/- 38 ng/uL	1552 +/- 57 ng/uL	1.84
SCC9	461 +/- 41 ng/uL	1499 +/- 61 ng/uL	1.81
SCC15	492 +/- 44 ng/uL	1523 +/- 52 ng/uL	1.82
SCC25	443 +/- 49 ng/uL	1531 +- 51 ng/uL	1.88
CAL27	455 +/- 51 ng/uL	1528 + 47 ng/uL	1.86
Average	454.6 +/- 44.6 ng/uL	1526 +/- 53.6 ng/uL	1.84
Range	422 - 492 ng/uL	1499 - 1552 ng/uL	1.81 - 1.88
